# Identification of Active Compounds of Mahuang Fuzi Xixin Decoction and Their Mechanisms of Action by LC-MS/MS and Network Pharmacology

**DOI:** 10.1155/2020/3812180

**Published:** 2020-05-23

**Authors:** Xiao Liang, Chang-Shun Liu, Ting Xia, Qing-Fa Tang, Xiao-Mei Tan

**Affiliations:** ^1^School of Traditional Chinese Medicine, Southern Medical University, Guangzhou, China; ^2^Guangdong Provincial Key Laboratory of Chinese Medicine Pharmaceutics, Guangzhou, China; ^3^Guangdong Provincial Engineering Laboratory of Chinese Medicine Preparation Technology, Guangzhou, China

## Abstract

The decoction is an important dosage form of traditional Chinese medicine (TCM) administration. The Mahuang Fuzi Xixin decoction (MFXD) is widely used to treat allergic rhinitis (AR) in China. However, its active compounds and therapeutic mechanisms are unclear. The aim of this study was to establish an integrative method to identify the bioactive compounds and reveal the mechanisms of action of MFXD. LC-MS/MS was used to identify the compounds in MFXD, followed by screening for oral bioavailability. TCMSP, BindingDB, STRING, DAVID, and KEGG databases and algorithms were used to gather information. Cytoscape was used to visualize the networks. Twenty-four bioactive compounds were identified, and thirty-seven predicted targets of these compounds were associated with AR. DAVID analysis suggested that these compounds exert their therapeutic effects by modulating the Fc epsilon RI, B-cell receptor, Toll-like receptor, TNF, NF-*κ*B, and T-cell receptor signaling pathways. The PI3K/AKT and cAMP signaling pathways were also implicated. Ten of the identified compounds, quercetin, pseudoephedrine, ephedrine, *β*-asarone, methylephedrine, *α*-linolenic acid, cathine, ferulic acid, nardosinone, and higenamine, seemed to account for most of the beneficial effects of MFXD in AR. This study showed that LC-MS/MS followed by network pharmacology analysis is useful to elucidate the complex mechanisms of action of TCM formulas.

## 1. Introduction

Allergic rhinitis (AR), an immunoglobulin E- (IgE-) mediated inflammatory disease, seriously impairs the quality of life of patients [1]. Epidemiological surveys show that AR affects more than 20% of the world's population and its incidence has progressively increased in developing countries [2]. Currently, treatments based on Western medicine alleviate the symptoms of AR but do not cure. Once drug treatments stop, the disease relapses [3]. Traditional Chinese medicine (TCM) has long been used as effective therapeutic interventions in Asia, particularly China [4]. TCM is primarily based on the use of compound formulas, utilizing a combination of herbs or their extracts for improved efficacy. Research into the active and effective compounds of TCM promotes the development and design of new therapeutic drugs.

The decoction is the main form of TCM administration. The identification of the chemical components in a decoction underpins research on the mechanism of action of TCM. Mahuang Fuzi Xixin decoction (MFXD), a classical Chinese herbal formula, is widely used to treat AR. MFXD consists of Mahuang (*Herba Ephedrae)*, Fuzi (*Radix aconiti lateralis praeparata*), and Xixin (*Radix et Rhizoma Asari*), which are boiled in water at specific proportions before administration. Pharmacological studies have shown that MFXD exerts anti-inflammatory and antiallergic effects by preventing the release of mediators from macrophages and mast cells and inhibiting the production of interferon gamma and interleukin (IL)-4 [5, 6]. MFXD also suppresses Th2 cytokine production and regulates the balance of Th1 and Th2 responses [7]. Using collected compounds and targets from databases and references, Tang constructed a network that shows the interactions of components and targets and verified the pharmacological activity of four components (salsolinol, pseudoephedrine, dibutyl phthalate, and herbacetin) of MFXD *in vitro* [8]. However, to our knowledge, there have been no comprehensive studies of the compounds in the decoction prepared from MFXD and their potential therapeutic mechanism in AR.

Network pharmacology is a new field that integrates pharmacological information, omics, and systems biology [9]. It is based on the concept that targeting multiple nodes in interconnected systems, rather than individual nodes, generates information for the identification of a drug with better efficacy and fewer adverse effects. TCM formulas commonly comprise a mixture of several herbs and ingredients that deliver synergistic effects by targeting multiple targets and pathways and modulating the links between pathways. Thus, network pharmacology, which elucidates interactions between multiple compounds and targets, is particularly suited for investigating the activities of TCM [10, 11]. Currently, drug discovery is rapidly evolving towards systematic and multipharmacological approaches to address the poor efficacy, loss of efficacy, and development of drug resistance when single compounds are used to target single therapeutic targets [12, 13]. Research is now focused on the simultaneous investigation of targets in the context of entire biological networks [14]. This network pharmacology approach to drug discovery and design may identify effective drugs from herbal medicines.

Therefore, the aim of this study was to identify the active compounds in the decoction prepared from MFXD to investigate their potential synergistic effects on cellular signaling pathways that are associated with AR. In this study, the combinatorial approach of LC-MS/MS followed by network pharmacology analysis was established to identify the active chemical compounds in the decoction prepared from MFXD and their potential pharmacological mechanism in AR. This research considers bioactive compounds, protein targets, protein-protein interactions, genes, and signaling pathways to elucidate the mechanisms responsible for the curative effects of MFXD in AR.

## 2. Materials and Methods

### 2.1. Materials

HPLC-grade acetonitrile and methanol were procured from Merck (Darmstadt, Germany). Formic acid was procured from Sigma-Aldrich (MO, USA). Other chemicals were of analytical grade.

The decocting pieces of Mahuang, Fuzi, and Xixin were purchased from Kangmei Pharmaceutical Co., Ltd. (Guangzhou, China) and met the standards of the Chinese Pharmacopeia. Voucher specimens (no. 20191117) were deposited at the authors' laboratory at Southern Medical University.

### 2.2. Preparation of MFXD

MFXD consisted of Mahuang, Fuzi, and Xixin at a weight ratio of 2 : 3 1. Mahuang was immersed in distilled water (15 times the total weight) for 30 min and boiled for 20 min. Fuzi and Xixin were then added to the suspension, which was simmered for another 90 min. The liquid extract obtained was concentrated to 1.52 g/mL under reduced pressure.

### 2.3. LC-MS/MS Analyses

LC-MS/MS was carried out using a UPLC-Orbitrap-HRMS platform (Thermo Fisher) with a Waters ACQUITY UPLC HSS T3 Column (100 mm × 2.1 mm, 1.8 *μ*m). The mobile phases consisted of acetonitrile (A) and 0.1% aqueous formic acid (v/v) (B) using a gradient elution of 0% A at 0−1 min, 0−20% A at 1−2 min, 20−50% A at 2−12 min, 50−95% A at 12−15 min, and 95−100% A at 15−20 min. The flow rate was set at 0.4 mL/min, and the column temperature was maintained at 30°C. The injection volume was 5 *μ*L. The electrospray ionization source was set to positive and negative modes. The mass range scanned was 100−1000 m/z. MS data were collected with Thermo Xcalibur software (version 4.0).

### 2.4. Construction of the Chemical Ingredient Database

We processed the chromatogram by matching the data with an in-house Orbitrap Traditional Chinese Medicine Library (OTCML), which provided a list of compounds with their formulas and MS/MS fragment modes. Based on errors less than 5 ppm and MS/MS fragment matching, we identified compounds in decoction prepared from MFXD.

### 2.5. Oral Bioavailability (OB) Screening

OB is an important pharmacokinetic property used to assess the rate and percentage of an orally administered drug that has been absorbed into the blood circulation to produce pharmacological effects. Important parameters of the identified compounds, such as Caco-2 cell permeability, human intestinal absorption (HIA), and OB limits F (F-20%, F-30%), were screened using ADMETlab, a platform for systematic ADMET evaluation based on a comprehensively collected ADMET database [15].

### 2.6. Potential AR-Associated Targets of the Compounds in MFXD

Predicting whether a compound interacts with intended targets is a critical phase of drug discovery [16]. The targets of compounds in MFXD were obtained from the Traditional Chinese Medicine System Pharmacology (TCMSP, http://lsp.nwu.edu.cn/tcmsp.php) and BindingDB (https://www.bindingdb.org/bind/index.jsp) databases. After the deletion of redundant hits, the remaining protein targets were standardized by their ID and gene symbols in the UniProtKB database (http://www.uniprot.org/) [17].

Information on AR-associated targets was retrieved from the therapeutic target disease (http://db.idrblab.net/ttd/), DrugBank (https://www.drugbank.ca), and DisGeNET (https://www.disgenet.org/) databases and standardized using the UniProtKB database.

### 2.7. Construction of the Target Protein-Protein Interaction (PPI) Network

To determine whether the therapeutic targets of MFXD are associated with AR, we intersected the drug targets and disease targets to obtain the common targets, which were considered potential therapeutic targets.

The common target proteins were used to construct the PPI network on the Search Tool for the Retrieval of Interacting Genes/Proteins (STRING) 11.0 platform (https://string-db.org/) using the minimum required interaction score of 0.400. Subsequently, the topological property of the PPI network was analyzed using plugins in Cytoscape 3.7.2. Genes were ranked to define hub genes using cytoHubba with the maximal clique centrality (MCC) algorithm. The module was extracted by MCODE using a node score cutoff of 0.2 and K-core of 2.

### 2.8. Target Pathway and Enrichment Analysis

To analyze the gene ontology (GO) functional annotations and Kyoto Encyclopedia of Genes and Genomes (KEGG) pathway enrichment of genes and their roles in signal transduction, the Database for Annotation, Visualization, and Integrated Discovery (DAVID 6.8) was employed. DAVID is an online analytical program that provides a comprehensive set of functional annotation tools to explore the biological functions of genes from gene lists [18]. DAVID can identify and describe the biological processes, cellular components, molecular functions, and pathways that are associated with genes of interest.

### 2.9. Construction of Compound-Target-Pathway Networks

To understand the underlying mechanism of MFXD in AR, a large-scale combinatorial network was established by integrating data obtained on drug information, drug-target interaction, and target-related pathway interactions. Through the hierarchical network, we characterized the relationships and pathways targeted by MFXD in AR.

## 3. Results and Discussion

### 3.1. Analysis of Chemical Compounds from MFXD

**Figure 1:**
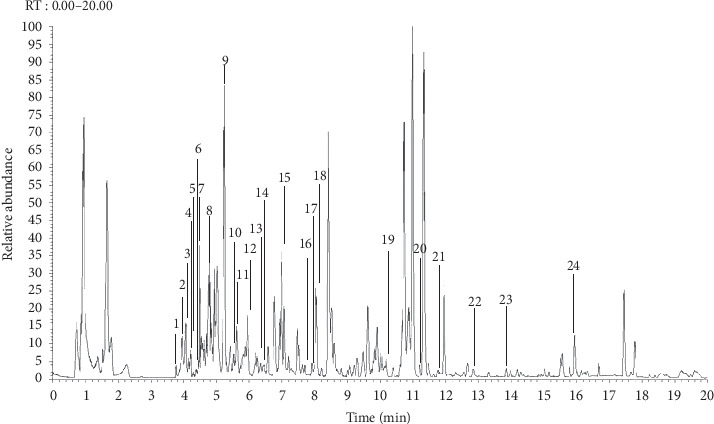
Total ion current chromatogram of LC-MS/MS of MFXD.

The total ion chromatogram of MFXD is shown in Figure 1. This was matched with the OTCML database, jointly constructed by Thermo Scientific and Tsinghua University as a reference for compound identification. Using electrostatic field orbital trap high-resolution MS for fragment MS acquisition, more than 1,200 reference compounds of TCM have been collected in this database. More than 7,000 second-order MS images of the highest quality are available, enabling the rapid and accurate characterization of TCM components and natural products. A total of 24 compounds in MFXD were identified through chromatogram matching.  Table 1 shows retention time, experimental and calculated m/z values, molecular formulas, errors in parts per million (ppm), and the major MS/MS fragments. The mass error of all identified compounds was less than 5 ppm.

If a compound is poorly absorbed following oral administration, the pharmacological effects may not be realized, even if the compound has potent effects on the pharmacological target *in vitro* [19]. In this study, Caco-2, OB limits F (20% and 30% bioavailability), and HIA were used to assess the components identified in MFXD. The results from OB and MS were combined to select compounds that were considered to be orally bioactive in the decoction prepared from MFXD.  Table 2 presents the ADMET properties of the 24 components identified in MFXD. Most of the compounds showed favorable absorption properties.

### 3.2. Construction and Analysis of the Component-Target Interaction Network

The TCMSP database contains 499 Chinese herbs with 19,384 compounds, 3,311 targets, and 837 associated diseases [20]. BindingDB is an experimental protein-small molecule database for virtual compound screening based on maximal chemical similarity and support vector machine methods [21]. We screened the targets from the TCMSP and BindingDB databases for the 24 active compounds. A total of 198 targets were obtained after the deletion of redundant hits (113 from TCMSP and 138 from BindingDB).

**Figure 2:**
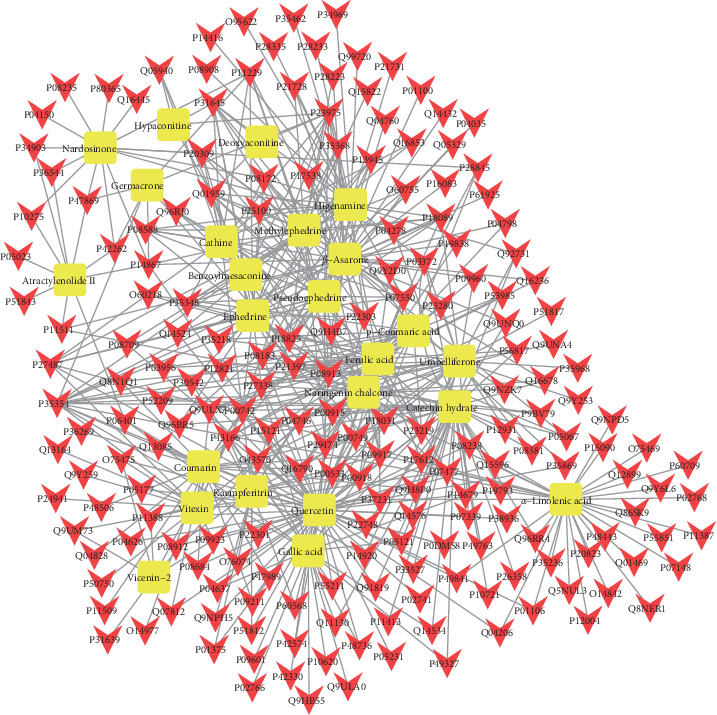
Compound-compound target network of MFXD consists of 24 compounds and 198 compound-target nodes (the yellow rectangles are the compounds and the red triangle targets are compound targets).

To visualize the relationship between compounds and their targets, we constructed a compound-target interaction network (Figure 2). This network had a density of 0.022 with a characteristic path length of 3.396 and an average number of 4.918 neighbors. In the compound-target network, multiple compounds could act on the same target protein and a single compound could be associated with multiple target proteins. For instance, prostaglandin G/H synthase 2 (PTGS2-P35354) and sodium-dependent noradrenaline transporter (SLC6A2-P23975) represented the hub nodes with a high degree of distribution, whereas peripheral nodes, such as gallic acid, interacted with the progesterone receptor (PGR-P06401) and represented a lower degree of distribution. These results were consistent with the common characteristics of TCM, in which multiple compounds and multiple targets are observed.

### 3.3. Construction and Analysis of the Target PPI Network

**Figure 3:**
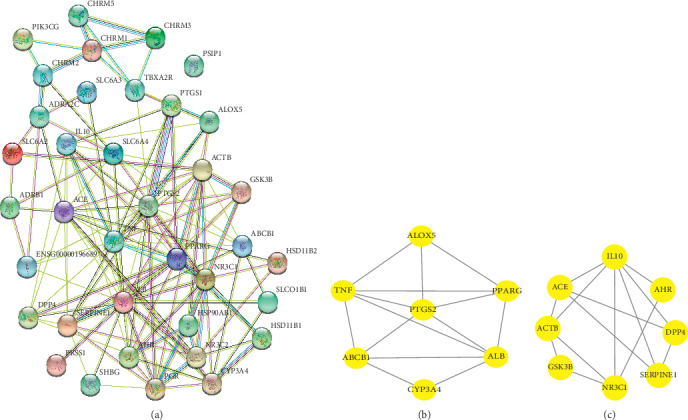
The protein-protein interaction (PPI) network and significant module constructed by the STRING database and Cytoscape 3.7.2. Node, target proteins; lines, interactions between proteins. (a) PPI of the common targets related to AR interacting with MFXD molecules. (b, c) Significant module selected from PPI.

Through the comparative analysis of 198 component targets and AR-related disease targets, we identified 37 common potential targets for MFXD in AR (Table S1). The STRING database is a commonly used tool for predicting PPI and producing integrated and objective association networks [22]. Using the common protein targets as input for network visualization, a diversified PPI network was created (Figure 3(a)). The network systematically summarized the interactions of MFXD targets associated with AR treatment. PSIP1 was not analyzed in the PPI network, as it does not interact with other proteins. The network shows viable protein target nodes (*n* = 36) connected by edges (*n* = 135) with an average node degree of 7.5 and average local clustering coefficient of 0.548. The PPI enrichment *p* value was less than 1.0 e^−16^; thus, proteins have more interactions among themselves than would be expected for a random set of proteins of similar size drawn from the genome. Such a significant enrichment indicated that the proteins are at least partially biologically connected as a group.

The genes ranked by the MCC algorithm were selected using the cytoHubba plugin. The predicted top 10 contributing hub genes were *TNF, PPARG, ALB, PTGS2, IL-10, ACTB, NR3C1, ACE, SERPINE1,* and *AHR,* which were considered crucial targets of MFXD against AR.

Two significant modules were selected from the PPI network by MCODE (Figures 3(b) and 3(c)). For module 1, the genes were significantly enriched in the nuclear factor-*κ*B (NF-*κ*B) signaling pathway and tumor necrosis factor (TNF) signaling pathway. Cluster 2 genes were significantly enriched in the T-cell receptor signaling pathway. These data suggested that MFXD likely acts on AR through all three signaling pathways.

### 3.4. DAVID Pathway Analysis

**Figure 4:**
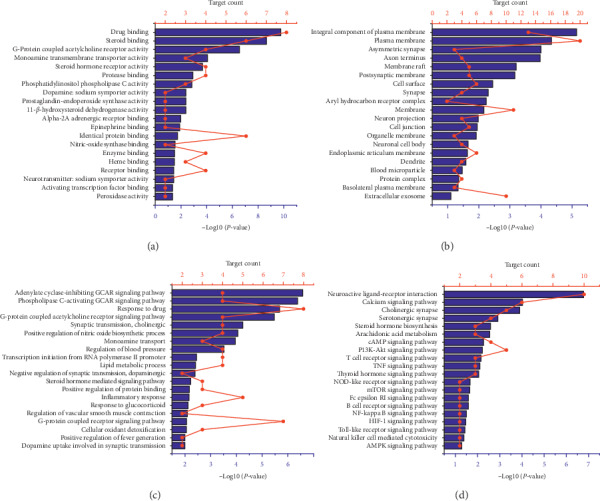
GO enrichment and KEGG pathway analysis for targets of MFXD constructed by DAVID 6.8. The order of importance was ranked from the top to bottom by −Log10 (*P* value) with bar charts. The number of targets sticks into each term with line charts. Go analysis was done under the categories of (a) molecular function, (b) cellular component, (c) biological process, and (d) KEGG enrichment pathway. GCAR stands for G-protein-coupled acetylcholine receptor.

To further investigate the multiple mechanisms of MFXD at a systematic level, 37 common genes were uploaded into DAVID 6.8. Functional association clustering analysis discovered 14 annotation clusters, and the highest enrichment score was 4.07. The top 20 terms in molecular function, cellular component, and biological process are presented in Figures 4(a)–4(c), respectively.

GO enrichment analysis showed that the target genes were expressed in the plasma membrane, cell surface, cell membrane, and other cell compartments. At the molecular level, the target genes were involved in drug binding, enzyme binding, G-protein acetylcholine receptor activity, and steroid binging. At the cellular level, they were related to cell apoptosis, proliferation, and migration. Moreover, biological processes were also enriched with inflammation-related terms. AR is a chronic inflammatory disease that involves the release of various inflammatory mediators [1]. Therefore, the inhibition of the inflammatory response is a therapeutic strategy for AR.

**Figure 5:**
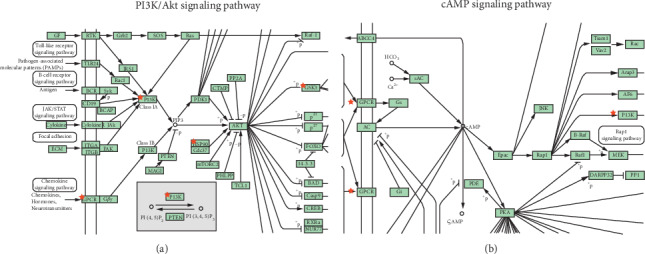
DAVID pathway analysis. (a) PI3K/Akt signaling pathway. (b) cAMP signaling pathway. The stars indicate the targets where the molecules interact.

KEGG pathway annotation showed that 31 of the 37 (86.1%) potential target genes were enriched and involved in 153 pathways associated with the immune system, cardiovascular system, cancer, inflammation, and diabetes. As AR is an allergic disease, pathways that are associated with the immune system and inflammation were selected (Figure 4(d)). For example, the Fc epsilon RI, B-cell receptor, T-cell receptor, and Toll-like receptor signaling pathways are involved in the regulation of the immune system and the TNF and NF-*κ*B signaling pathways are involved in inflammation. We also identified the PI3K/AKT, cAMP, and AMPK signaling pathways in this analysis. The PI3K/AKT signaling pathway is important for cell growth, differentiation, metabolism, survival, and apoptosis [23]. Recent research has revealed that the PI3K-AKT signaling pathway regulates mast cell activity and is modulated in AR [24, 25]. Moreover, the central and peripheral regulation of energy homeostasis relies on the cAMP signaling pathway [26]. KEGG pathway analysis showed that the active components of MFXD act on the gene nodes within the PI3K/AKT and cAMP signaling pathways (Figure 5). These data are consistent with the multiple effects that TCM has on different signaling and cellular pathways [27].

Comparative analysis of these KEGG pathways revealed that, among the 37 potential target genes identified, genes that were repeatedly associated with these pathways were *PIK3CG, TNF, PTGS2, CHRM2, CHRM1, GSK3B, ADRB1, CHRM5, CHRM3,* and *HSP90AB1*.

### 3.5. Compound-Target-Pathway Network Analysis of MFXD

**Figure 6:**
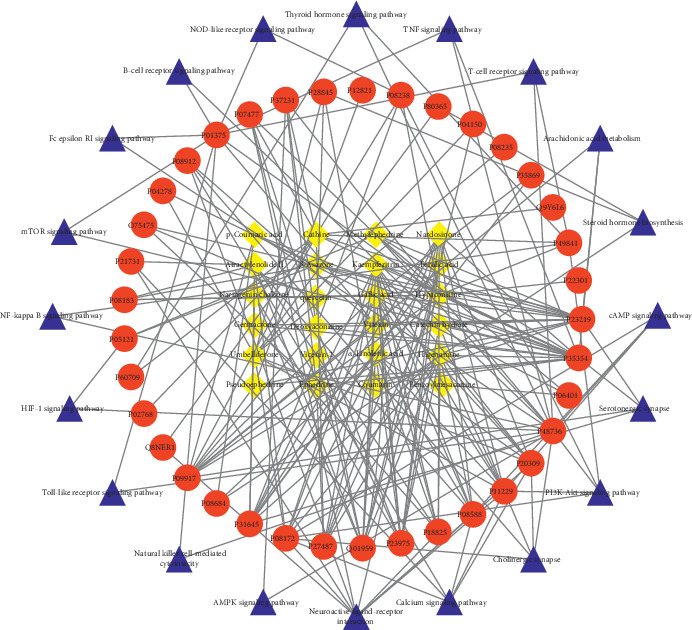
The pharmacological network (yellow diamonds, compounds; red ellipse, targets; blue triangle, pathway).

Chinese herbal formulas can contain dozens, or even hundreds, of compounds, and each compound can act on one or multiple targets to exert synergistic therapeutic effects [28]. To directly explore potential synergistic relationships, a compound-target-pathway combination network was constructed (Figure 6). This network revealed that MFXD contains multiple compounds that have multiple targets within multiple pathways that are associated with AR treatment. For example, quercetin acted on 14 targets (such as P48736, P22301, Q75475, and P08684) and gallic acid acted on three targets (P23219, P35354, and P48736). P48736 was associated with both quercetin and gallic acid. Using network topological analysis, the top 10 compounds that may make major contributions to AR treatment are presented in descending order: quercetin, pseudoephedrine, ephedrine, *β*-asarone, methylephedrine, *α*-linolenic acid, cathine, ferulic acid, nardosinone, and higenamine. Quercetin can stimulate the immune system, inhibit the release of histamine, decrease the production of proinflammatory cytokines, increase the synthesis of leukotrienes, restrain the formation of IgE antibody, and improve the Th1/Th2 balance by participating in multiple signal pathways [29]. All these mechanisms of action contribute to the anti-inflammatory and immunomodulatory properties of quercetin, which can be effectively utilized in the treatment of AR, as shown in an animal model [30]. Gallic acid alleviates nasal inflammation via the activation of Th1 and inhibition of Th2 and Th17 cells and has immunomodulatory effects [31]. *α*-Linolenic acid dampens AR through the eosinophilic production of 15-hydroxyeicosapentaenoic acid [32], and ephedrine has been used as a nasal wash for AR treatment [33].

Most of the compounds, such as kaempferitrin and pseudoephedrine, interact with PTGS2 (P35354). PTGS2 is responsible for the production of inflammatory prostaglandin E2, which inhibits T regulatory cell differentiation to induce AR-related inflammation [34]. We also discovered compounds that acted together on the targets P01375 and P48736 to regulate the Fc epsilon RI signaling pathway. Gallic acid was observed to interact with PIK3CG (P48736), a key protein that activates the B-cell receptor, T-cell receptor, and Toll-like receptor signal pathways. Finally, targets such as P48736, P01375, P48736, P01375, and P49841, which may provide protection against AR, interacted with most of the compounds.

## 4. Conclusion

Unlike Western medicines, TCMs are commonly prescribed as herbal formulas that contain a mixture of herbs. Each herb may contain many active ingredients that have single or multiple targets; thus, it is difficult to pinpoint the mechanisms of action of TCM. Network pharmacology shares the core concepts of the holistic philosophy of TCM and meets the requirements to treat complex diseases systematically [35]. However, in the traditional research of network pharmacology, the compounds are mostly collected from databases. Some compounds cannot be detected in the decoction, which may yield false positive results.

In this study, LC-MS/MS identification of compounds in the decoction followed by network pharmacology analyses provided insights into the mechanism of MFXD in the treatment of AR. In total, 24 bioactive compounds were identified in MFXD and 37 common targets were obtained and analyzed. The results indicated that MFXD was effective in the treatment of AR by regulating key pathways, including the Fc epsilon RI, B-cell receptor, Toll-like receptor, NF-*κ*B, T-cell receptor, PI3K-AKT, cAMP, and AMPK signaling pathways. Ten compounds in the decoction prepared from MFXD were identified as candidates that could target AR.

These results reduce the prediction range, increase the accuracy of the prediction results, and provide important information for further pharmacological investigations on MFXD. This method, LC-MS/MS for bioactive compound identification followed by network pharmacology analyses, can increase the understanding of the mechanisms of Chinese herbal formulas and promote drug research and development.

## Figures and Tables

**Table 1:** Results of LC-MS/MS analysis of MFXD.

No.	Retention time	Theoretical m/z	Measured m/z	Error ppm	Molecular formula	Major MS/MS fragments	Compound
1	3.78	170.0215	170.0211	−2.3526	C_7_H_6_O_5_	169.0135	Gallic acid
						125.0239	
						97.0291	
						79.0187	

2	4.02	151.0997	151.0992	−3.3091	C_9_H_13_NO	134.0965	Cathine
						117.0698	
						115.0543	

3	4.03	165.1154	165.1158	2.4226	C_10_H_15_NO	148.1024	Ephedrine
						133.0804	
						117.0619	

4	4.11	271.1208	271.1199	−3.3196	C_16_H_17_NO_3_	270.1133	Higenamine
						162.0554	
						107.0498	

5	4.12	165.1154	165.1149	−3.0282	C_10_H_15_NO	132.1010	Pseudoephedrine
						114.8160	
						91.1290	

6	4.30	179.1310	179.1316	3.3495	C_11_H_17_NO	180.1383	Methylephedrine
						162.1277	
						117.0700	
						72.0809	

7	4.48	594.1585	594.1578	−1.1781	C_27_H_30_O_15_	577.1541	Vicenin-2
						457.1118	
						379.0798	
						337.0703	

8	4.75	290.0790	290.0796	2.0684	C_15_H_14_O_6_	289.0715	Catechin
						245.0815	
						203.0708	
						109.0289	

9	5.25	578.1636	578.1623	−2.2485	C_27_H_30_O_14_	577.2036	Kaempferitrin
						431.0986	
						285.0396	

10	5.42	432.1057	432.1051	−1.3886	C_21_H_20_O_10_	433.1122	Vitexin
						415.1014	
						313.0701	
						283.0597	

11	5.57	164.0474	164.0482	4.8766	C_9_H_8_O_3_	163.0393	p-Coumaric acid
						119.0496	
						93.0341	

12	5.97	302.0427.	302.0492	2.1520	C_15_H_10_O_7_	245.0447	Quercetin
						152.0185	
						150.9975	

13	6.33	272.0685	272.0679	−2.2053	C_15_H_12_O_5_	271.0613	Naringenin chalcone
						151.0032	
						119.0497	
						93.0342	

14	6.35	162.0317	162.0312	−3.0858	C_9_H_6_O_3_	161.0237	Umbelliferone
						133.0288	
						78.0768

15	6.98	589.2887	589.2889	3.3939	C_31_H_43_NO_10_	540.2580	Benzoylmesaconine
						508.2311	
						105.0331	

16	7.67	218.1671	218.1665	−2.7502	C_15_ H_22_ O	219.1738	Germacrone
						201.0905	
						145.1008	
						135.1165	

17	7.83	146.0368	146.0365	−2.0543	C_9_H_6_O_2_	119.0488	Coumarin
						103.0538	
						91.0538	

18	8.00	194.0579	194.0581	1.0306	C_10_H_10_O_4_	193.0502	Ferulic acid
						177.0542	
						134.0363	

19	10.28	615.3044	615.3030	−2.2753	C_33_H_45_NO_10_	556.2893	Hypaconitine
						524.2627	
						338.1745	
						105.0333	

20	11.23	629.3200	629.3193	−1.1123	C_34_H_47_NO_10_	570.3052	Deoxyaconitine
						538.2784	
						105.0331	

21	11.83	232.1463	232.1457	−2.5846	C_15_H_20_O_2_	215.1791	Atractylenolide II
						159.1165	
						145.1009	
						95.0853	

22	12.84	250.1569	250.1560	−3.5977	C_15_H_22_O_3_	233.1522	Nardosinone
						191.1428	
						95.0852	
						71.0128	

23	13.88	208.1099	208.1094	−2.4026	C_12_H_16_O_3_	191.0700	*β*-Asarone
						176.0465	
						168.0779	

24	15.94	278.2246	278.2241	−1.7971	C_18_H_30_O_2_	109.1009	Linolenic acid
						95.0852	
						81.0696	

**Table 2:** ADME profile of the compounds from MFXD.

Compounds	Caco-2	HIA	F20	F30
Gallic acid	−5.767	0.431	0.673	0.616
Cathine	−4.783	0.877	0.851	0.869
Ephedrine	−4.956	0.902	0.853	0.879
Higenamine	−5.293	0.538	0.226	0.386
Pseudoephedrine	−4.956	0.902	0.853	0.879
Methylephedrine	−4.477	0.809	0.701	0.707
Vicenin-2	−6.624	0.226	0.447	0.253
Catechin	−6.495	0.400	0.488	0.404
Kaempferitrin	−6.364	0.399	0.584	0.325
Vitexin	−6.317	0.263	0.494	0.287
p-Coumaric acid	−4.892	0.745	0.699	0.536
Quercitrin	−6.469	0.155	0.515	0.242
Naringenin chalcone	−5.198	0.472	0.596	0.519
Umbelliferone	−4.601	0.797	0.534	0.441
Benzoylmesaconine	−6.087	0.301	0.332	0.41
Germacrone	−4.378	0.768	0.661	0.625
Coumarin	−4.142	0.848	0.288	0.257
Ferulic acid	−4.943	0.635	0.680	0.509
Hypaconitine	−5.513	0.343	0.265	0.350
Deoxyaconitine	−5.469	0.349	0.261	0.355
Atractylenolide II	−4.35	0.866	0.564	0.549
Nardosinone	−4.353	0.826	0.633	0.585
*β*-Asarone	−4.379	0.763	0.722	0.681
Linolenic acid	−4.729	0.805	0.568	0.396

## Data Availability

The data used to support the findings of this study are included within the supplementary materials.

## Supplementary Materials

Supplementary Materials Table S1: information on 37 therapeutic targets in MFXD acting directly on AR.
