# Supplementary material for: Identification of Active Compounds of Mahuang Fuzi Xixin Decoction and Their Mechanisms of Action by LC-MS/MS and Network Pharmacology
Source: Evid Based Complement Alternat Med. 2020 May 23;2020:3812180. doi: 10.1155/2020/3812180 (PMC7267872; doi:10.1155/2020/3812180)
Supplement: Supplementary Materials — Table S1: information on 37 therapeutic targets in MFXD acting directly on AR. [file 3812180.f1.docx]

Table S1 Information on 37 therapeutic targets in MFXD acting directly on AR

| No. | Entry | Protein names | Gene names |
| --- | --- | --- | --- |
| 1 | P23219 | Prostaglandin G/H synthase 1 | *PTGS1* |
| 2 | P35354 | Prostaglandin G/H synthase 2 | *PTGS2* |
| 3 | P06401 | Progesterone receptor | *PGR* |
| 4 | P48736 | Phosphatidylinositol 4,5-bisphosphate 3-kinase catalytic subunit gamma isoform | *PIK3CG* |
| 5 | P20309 | Muscarinic acetylcholine receptor M3 | *CHRM3* |
| 6 | P11229 | Muscarinic acetylcholine receptor M1 | *CHRM1* |
| 7 | P08588 | Beta-1 adrenergic receptor | *ADRB1* |
| 8 | P18825 | Alpha-2C adrenergic receptor | *ADRA2C* |
| 9 | P23975 | Sodium-dependent noradrenaline transporter | *SLC6A2* |
| 10 | Q01959 | Sodium-dependent dopamine transporter | *SLC6A3* |
| 11 | P27487 | Sodium-dependent dopamine transporter | *DPP4* |
| 12 | P08172 | Muscarinic acetylcholine receptor M2 | *CHRM2* |
| 13 | P31645 | Sodium-dependent serotonin transporter | *SLC6A4* |
| 14 | P08684 | Sodium-dependent serotonin transporter | *CYP3A4* |
| 15 | P09917 | Arachidonate 5-lipoxygenase | *ALOX5* |
| 16 | Q8NER1 | Transient receptor potential cation channel subfamily V member 1 | *TRPV1* |
| 17 | P02768 | Serum albumin | *ALB* |
| 18 | P60709 | Actin, cytoplasmic 1 | *ACTB* |
| 19 | P05121 | Plasminogen activator inhibitor 1 | *SERPINE1* |
| 20 | P08183 | Plasminogen activator inhibitor 1 | *ABCB1* |
| 21 | P21731 | Thromboxane A2 receptor | *TBXA2R* |
| 22 | O75475 | PC4 and SFRS1-interacting protein | *PSIP1* |
| 23 | P04278 | Sex hormone-binding globulin | *SHBG* |
| 24 | P08912 | Muscarinic acetylcholine receptor M5 | *CHRM5* |
| 25 | P01375 | Muscarinic acetylcholine receptor M5 | *TNF* |
| 26 | P07477 | Trypsin-1 | *PRSS1* |
| 27 | P37231 | Peroxisome proliferator-activated receptor gamma | *PPARG* |
| 28 | P28845 | Corticosteroid 11-beta-dehydrogenase isozyme 1 | *HSD11B1* |
| 29 | P12821 | Angiotensin-converting enzyme | *ACE* |
| 30 | P08238 | Heat shock protein HSP 90-beta | *HSP90AB1* |
| 31 | P80365 | Corticosteroid 11-beta-dehydrogenase isozyme 2 | *HSD11B2* |
| 32 | P04150 | Glucocorticoid receptor | *NR3C1* |
| 33 | P08235 | Mineralocorticoid receptor | *NR3C2* |
| 34 | P35869 | Aryl hydrocarbon receptor | *AHR* |
| 35 | Q9Y6L6 | Solute carrier organic anion transporter family member 1B1 | *SLCO1B1* |
| 36 | P49841 | Glycogen synthase kinase-3 beta | *GSK3B* |
| 37 | P22301 | Interleukin-10 | *IL-10* |
